# Supplementary material for: Attentional Ptycho-Tomography (APT) for three-dimensional nanoscale X-ray imaging with minimal data acquisition and computation time
Source: Light Sci Appl. 2023 May 30;12:131. doi: 10.1038/s41377-023-01181-8 (PMC10227032; doi:10.1038/s41377-023-01181-8)
Supplement: Supplementary file 1 — Supplementary Information [file 41377_2023_1181_MOESM1_ESM.pdf]

Supplementary Information for

Attentional Ptycho-Tomography (APT) for three-dimensional nanoscale X-ray imaging with minimal data acquisition and computation time

Iksung Kang[1,‡,†], Ziling Wu[2,¶,†], Yi Jiang[3], Yudong Yao[3,§], Junjing Deng[3], Jeffrey Klug[3], Stefan Vogt[3], and George Barbastathis[2,4]

[1]Department of Electrical Engineering and Computer Science, Massachusetts Institute of Technology, Cambridge, Massachusetts 02139, USA

[2]Department of Mechanical Engineering, Massachusetts Institute of Technology, Cambridge, Massachusetts 02139, USA

[3]Argonne National Laboratory, Lemont, Illinois 60439, USA

[4]Singapore-MIT Alliance for Research and Technology (SMART) Centre, 1 CREATE Way, Singapore 138602

[‡]Present address: Department of Molecular and Cell Biology, University of California, Berkeley, California 94720, USA

[¶]Present address: Singapore-MIT Alliance for Research and Technology (SMART) Centre, 1 CREATE Way, Singapore 138602

[§]Present address: Center for Transformative Science, ShanghaiTech University, Shanghai 201210, China

[†]These authors contributed equally to this work.

[*]To whom correspondence should be addressed; E-mail: gbarb@mit.edu.

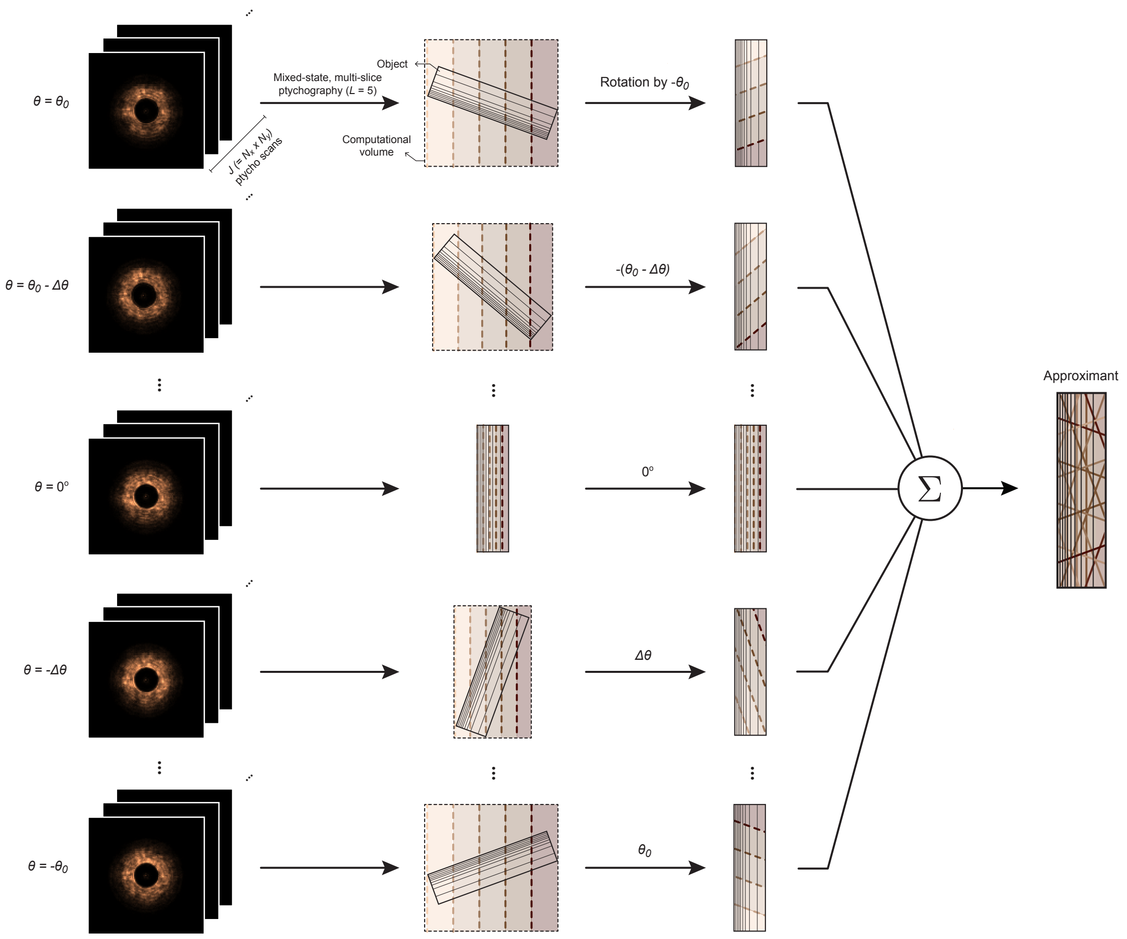

Figure S1. Detailed visualization of pre-processor. Our pre-processor serves as an approximate inversion operator acting upon diffraction intensities to derive the Approximant. J_n: the number of ptycho-scans for the n-th tomo-scan, and L: the number of slices assumed for the mixed-state, multi-slice ptychography algorithm. Rotation operations involve cubic interpolation and resampling of the estimates to the upright coordinate system.

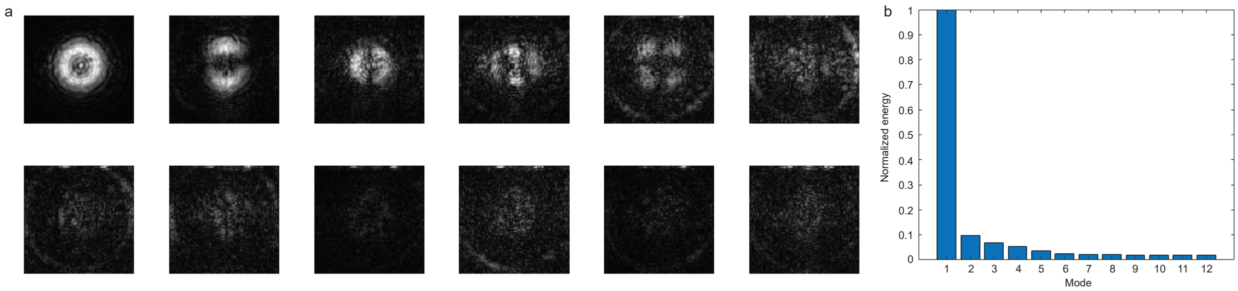

Figure S2. Coherent modes of synchrotron X-rays. (a) 12 coherent modes of the synchrotron X-ray illumination probe. **(b)** Normalized energy distribution of the probe modes.

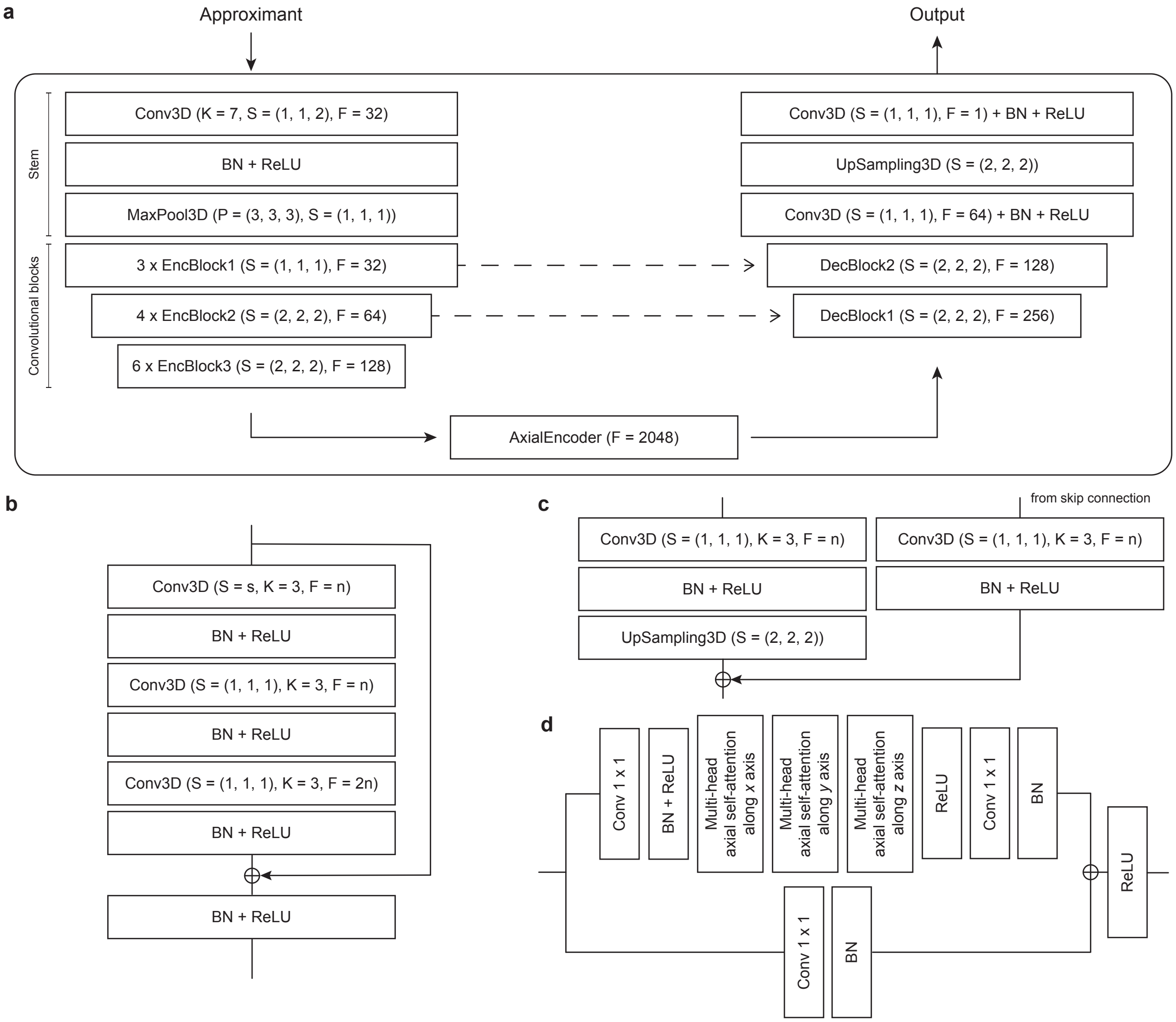

Figure S3. Machine learning architecture. (a) Tensorial diagram of our machine learning architecture (APT). **(b)** Detailed structure of EncBlock $(S = s,\ F = n)$, **(c)** DecBlock $(S = s,\ F = n)$ and **(d)** AxialEncoder $(F = n)$ (S: strides, K: kernel size, F: filters.) Scripts are publicly available at https://github.com/iksungk/APT.

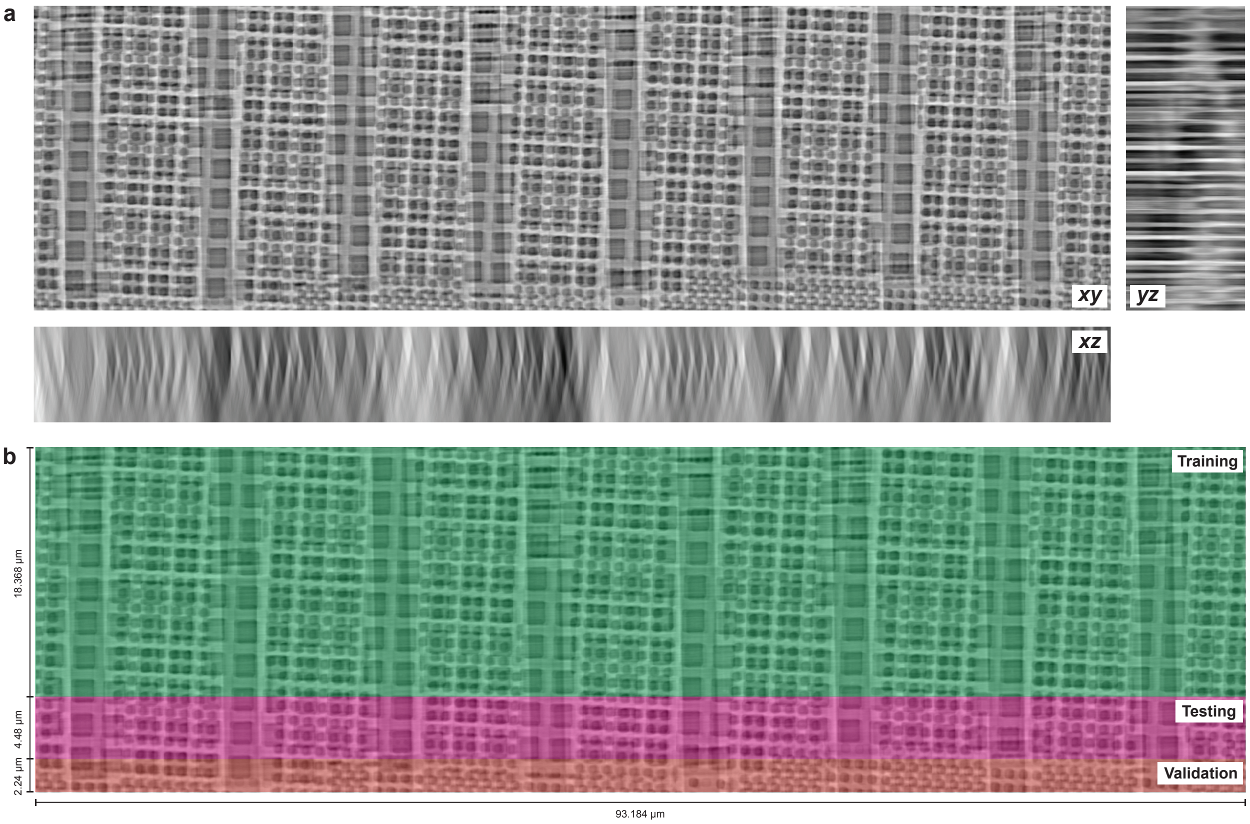

Figure S4. Approximant as input to the machine learning framework. (a) The figure shows the Approximant in *xy*, *xz*, and *yz* planes. **(b)** The Approximant is split into two non-overlap portions for training and testing/validation.

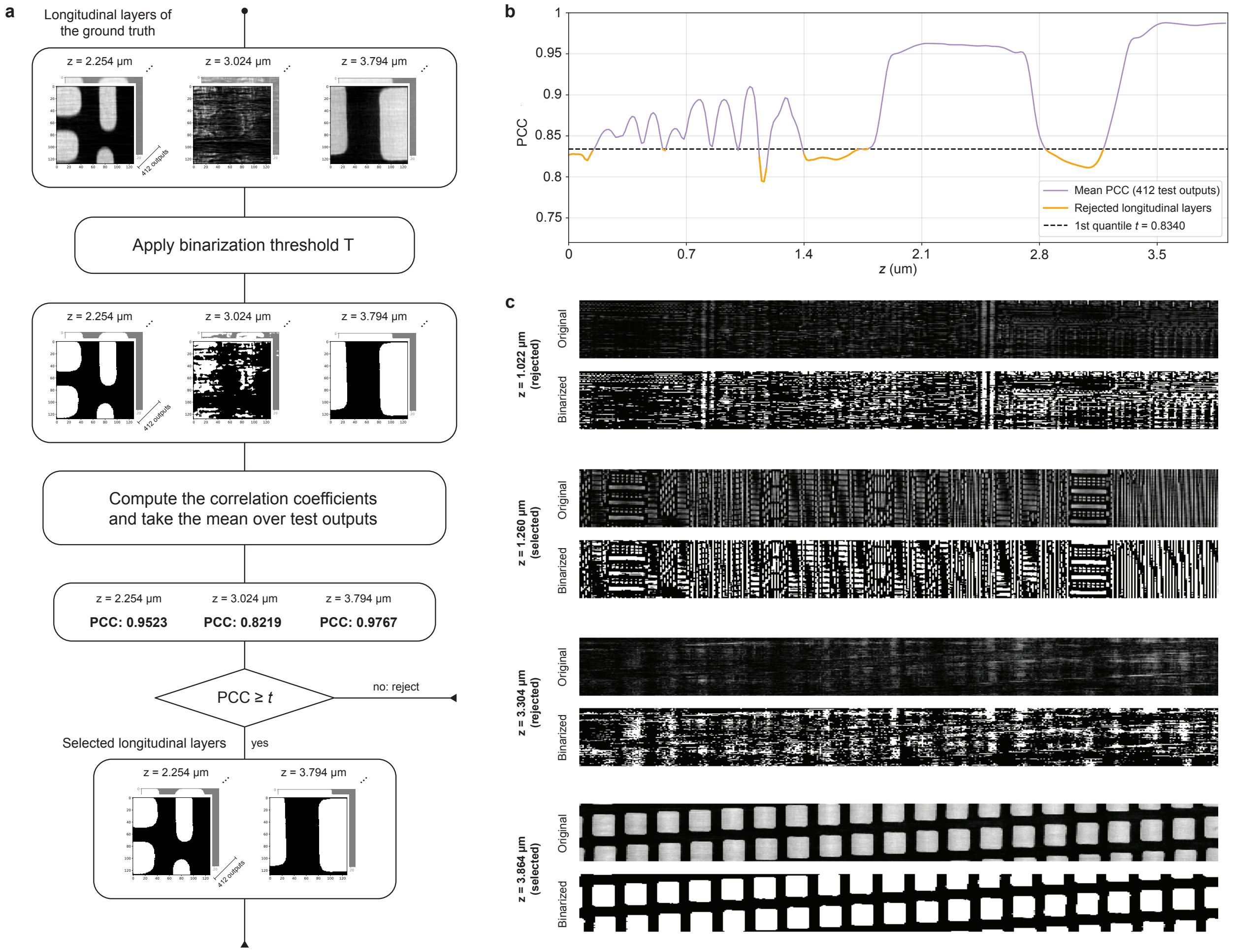

Figure S5. Additional consideration for the ground truth. (a) The figure gives detailed explanations on binarization of the ground truth (or gold standard) and exclusion of its ambiguous layers. **(b)** The ground truth layers with mean PCC over the test volume below $t = 0.8340$, *i.e.* the first quantile, are excluded from discussion. **(c)** The figure provides some of the selected and rejected ground truth layers over half the test volume ($4.48 \times 93.18 \times 3.92$ μm^3).

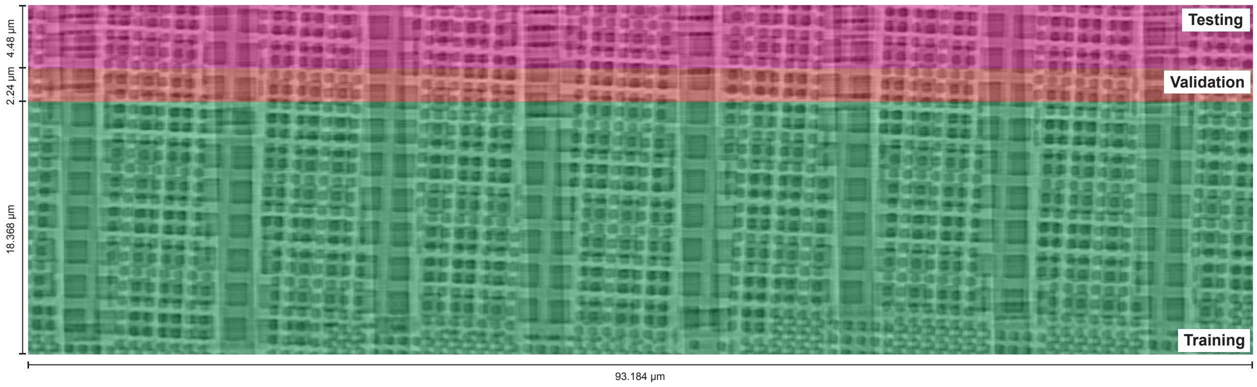

Figure S6. Different region division of the Approximant to prepare for training, validation, and testing datasets with no overlap. Results are shown in Figs. S7 and S8.

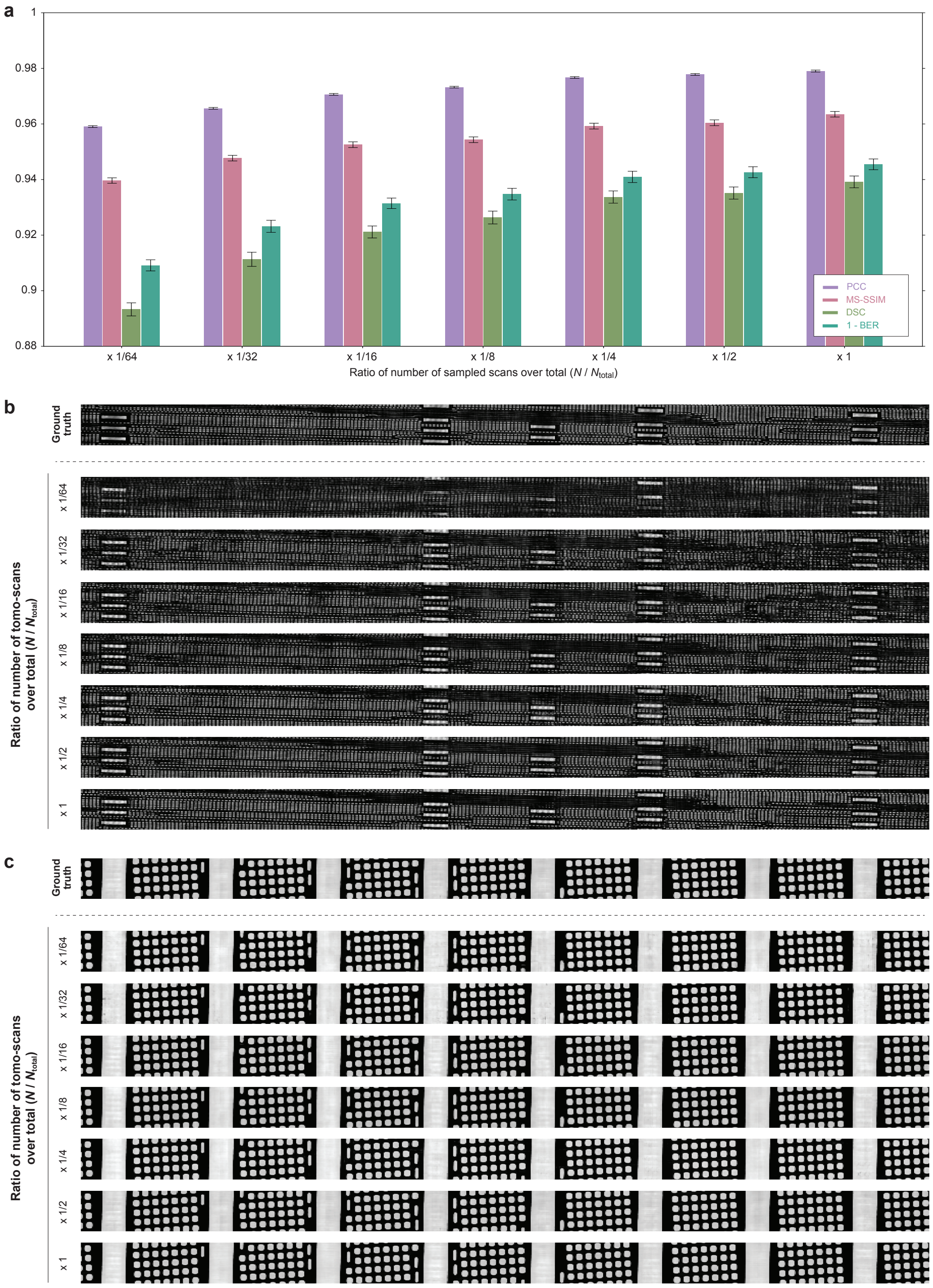

Figure S7. Quantitative and qualitative comparisons by sweeping the parameter of the number of tomo-scans (*N*). (a) The result of the quantitative comparison, where we determined that N* is 28.02, is comparable to the value of $N^* = 28.89$ presented in the main manuscript. **(b, c)** The qualitative comparisons of the reconstructed images under different scenarios are depicted at two distinct depths.

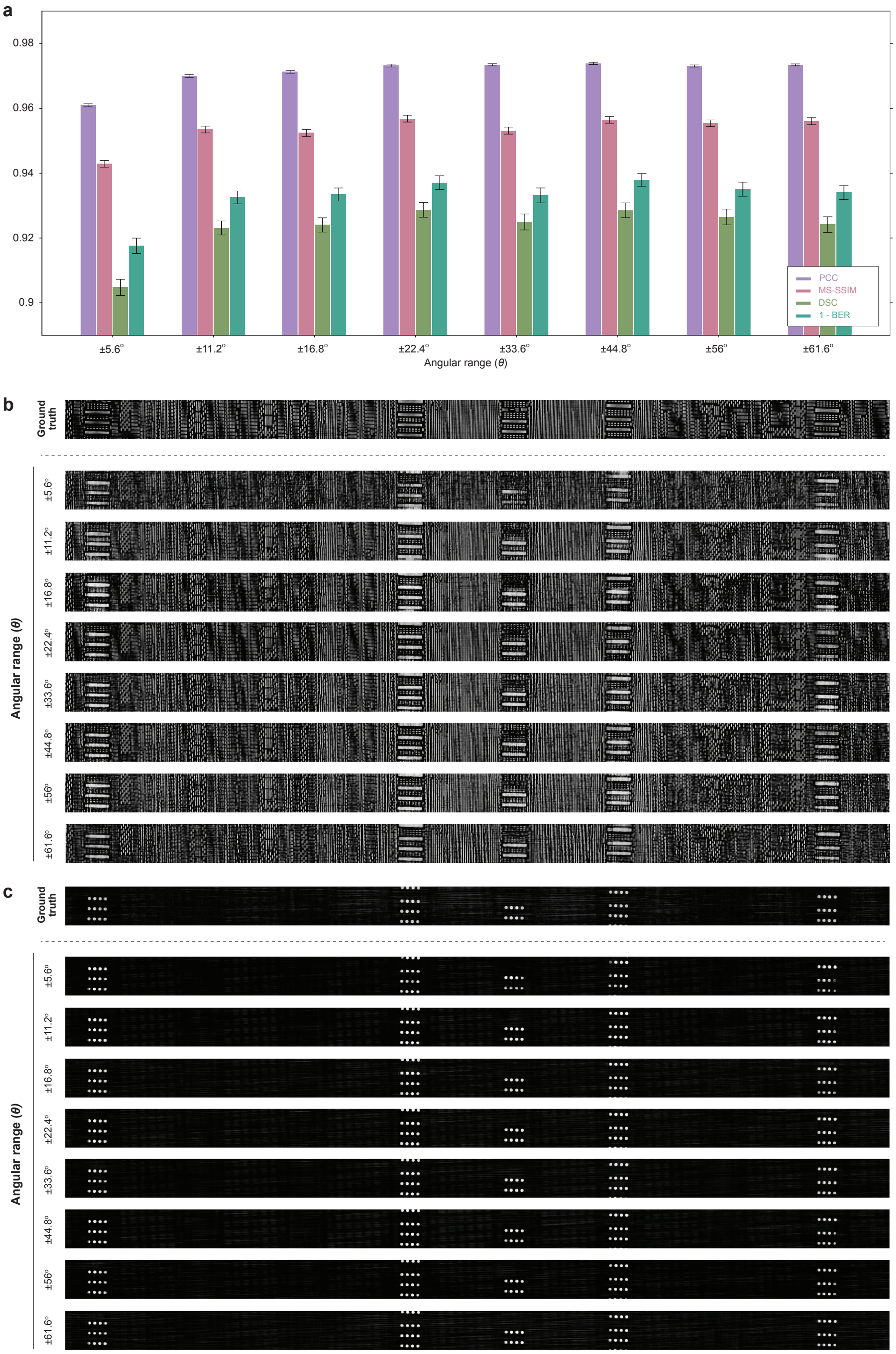

Figure S8. Quantitative and qualitative comparisons by sweeping the parameter of the angular scanning range. (a) The result of the quantitative comparison, where we determined that θ^* is $\pm 12.5°$, is in proximity to the value of $\theta^* = \pm 16.93°$ presented in the main manuscript. **(b, c)** The qualitative comparisons of the reconstructed images under different scenarios are illustrated at two different depths.

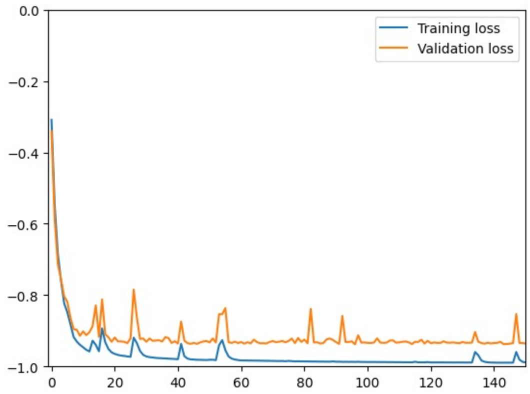

Figure S9. One example of training and validation loss curves. The two loss curves remained close to each other, indicating no overfitting or underfitting occurred during the training process.

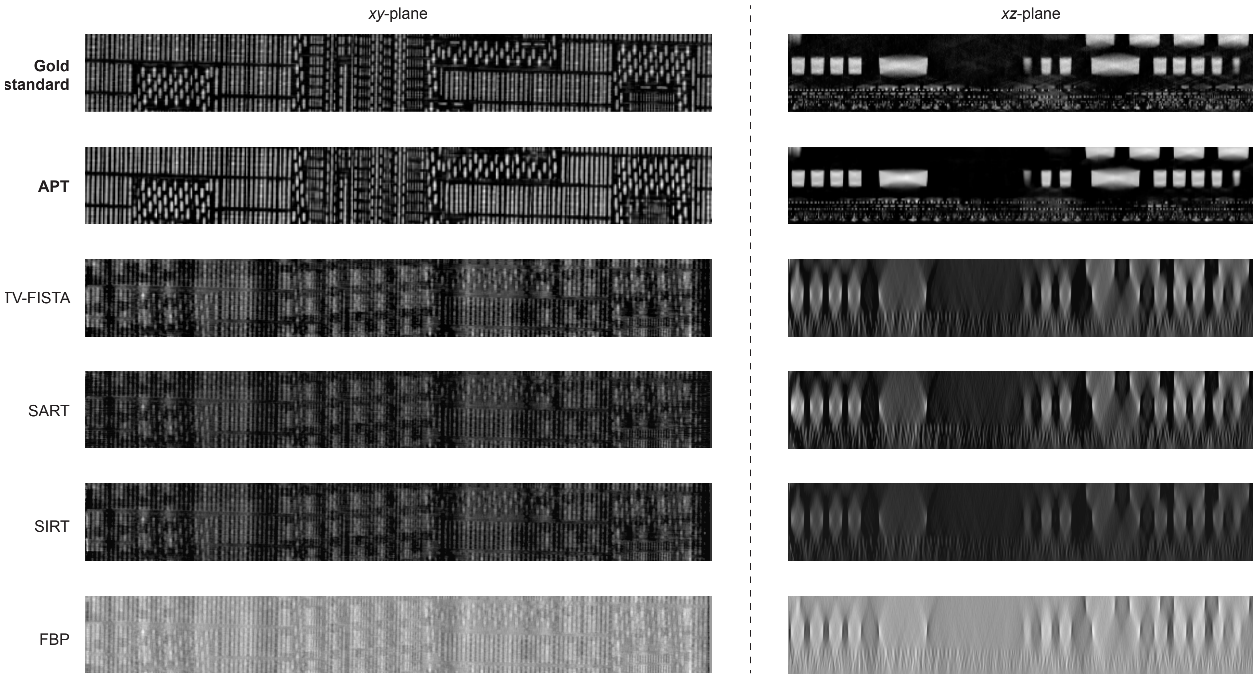

Figure S10. Qualitative comparison among reconstructions using different methods. TV-FISTA [1] was implemented using the tvtomo plugin under the TomoPy environment [2]. We set the TV regularization parameter to 10^{-6} with an additional minimum constraint, ran 50 iterations of FISTA update process, and reconstructed the ICS over 100 epochs. For SIRT and SART reconstructions, we ran 100 iterations of the algorithms. Our findings indicate that TV-FISTA is effective in reducing artifacts caused by sparse sampling, but it may not be sufficient to address the missing cone issue resulting from highly limited angular sampling, which could potentially be alleviated by supervised approaches, as mentioned in [3, 4].

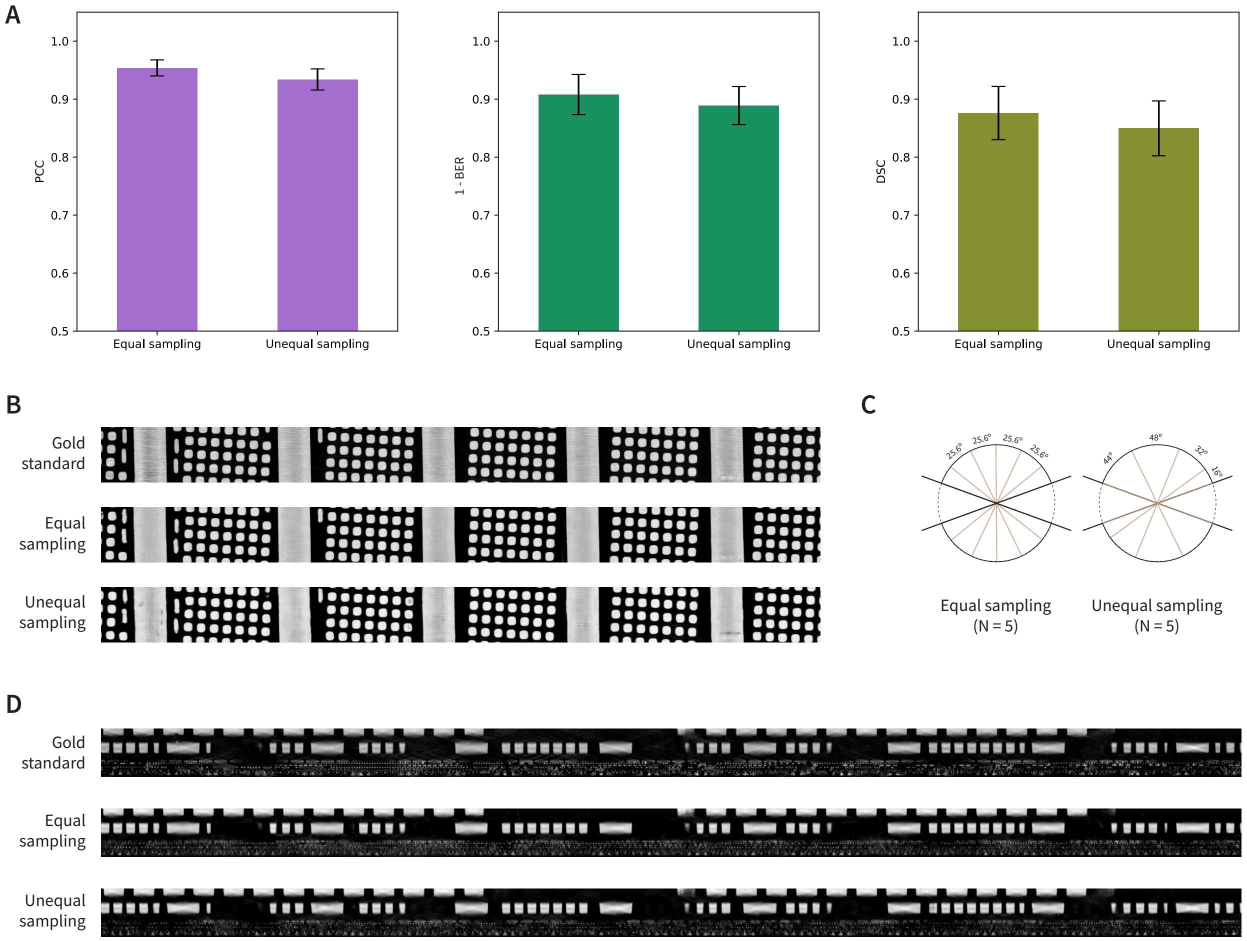

Figure S11. Differences between the reconstructions achieved using equal and unequal sampling schemes, both quantitatively and qualitatively. (a) The quantitative comparison was conducted using four different metrics, including the Pearson correlation coefficient (PCC), bit-error rate (BER), and Dice-Sørensen coefficient (DSC). **(b, d)** The qualitative comparison was carried out by comparing the reconstructions along the *xz* and *yz* planes. **(c)** Different angular sampling schemes. The performance with unequal angular sampling is slightly inferior to that of equal angular sampling, but the difference between the two is not significant.

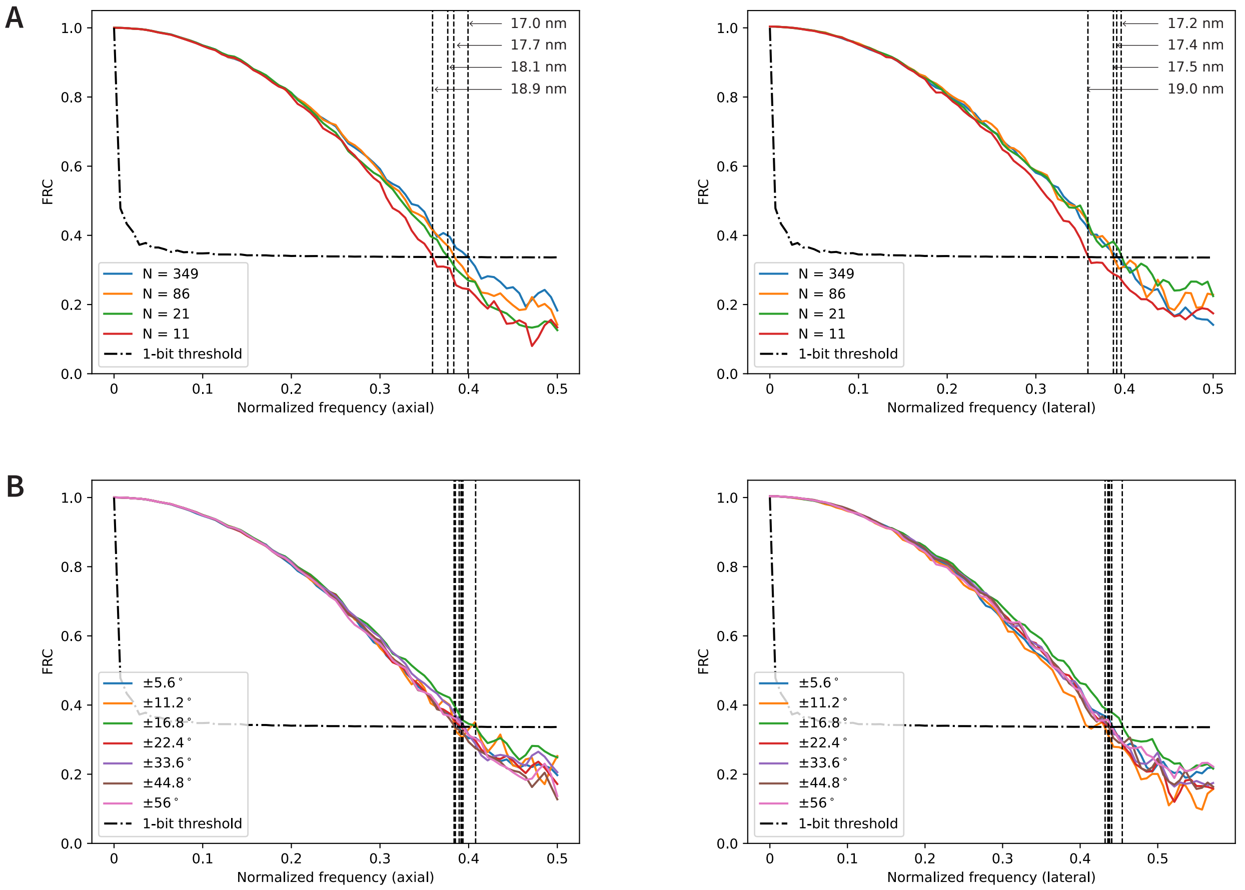

C

	±5.6°	±11.2°	±16.8°	±22.4°	±33.6°	±44.8°	±56°
**Axial**	17.5 nm	17.7 nm	16.7 nm	17.4 nm	17.3 nm	17.7 nm	17.3 nm
**Lateral**	17.6 nm	18.0 nm	17.1 nm	17.8 nm	17.7 nm	17.8 nm	17.6 nm

Figure S12. Resolution analysis. (a) Fourier ring correlation (FRC) curves of reconstructions with different number of tomo-scans with estimated axial and lateral resolutions. **(b)** FRC curves of reconstructions with different angular range of tomo-scans. **(c)** Estimated axial and lateral resolutions based on the curves in (b). FRC curves are derived based on one-image FRC [5].

Type	Time per tomo-scan (s)
Data acquisition (t_{DAQ})	23.04
Ptychographic reconstruction (t_{ptycho})	21.43
Data loading of a ptycho reconstruction to RAM (t_{load})	15.66
Rotation of a ptycho reconstruction to the upright coordinates (t_{rotate})	37.50
Generation of a machine learning (ML) reconstruction (t_{ML})	45

Table S1. Data acquisition and computation time when reducing the number of tomo-scans. Total data acquisition and computation time becomes $T = N \times (t_{\mathrm{DAQ}} + t_{\mathrm{ptycho}} + t_{\mathrm{rotate}} + t_{\mathrm{load}}) + t_{\mathrm{ML}}$ for $N = 5, 11, 21, 43, 86, 174, 349$.

Type	Time per tomo-scan (s)
Data acquisition (t_{DAQ})	23.04
Ptychographic reconstruction (t_{ptycho})	21.43
Data loading of a ptycho reconstruction to RAM (t_{load})	15.66
Rotation of a ptycho reconstruction to the upright coordinates (t_{rotate})	12.85, 14.98, 17.11, 19.24, 23.50, 27.76, 32.02
Generation of a machine learning (ML) reconstruction (t_{ML})	45

Table S2. Data acquisition and computation time when limiting the angular range of N^* tomo-scans. Total data acquisition and computation time becomes $T = N \times (t_{\mathrm{DAQ}} + t_{\mathrm{ptycho}} + t_{\mathrm{rotate}} + t_{\mathrm{load}}) + t_{\mathrm{ML}}$ for $\theta = [\pm 5.6°,\ \pm 11.2°\ \pm 16.8°,\ \pm 22.4°,\ \pm 33.6°, \pm 44.8°,\ \pm 56°]$.

References

1. Chambolle, A. & Pock, T., “A first-order primal-dual algorithm for convex problems with applications to imaging,” *J. mathematical imaging vision* 40, 120–145 (2011).
2. Pelt, D. M. et al., “Integration of tomopy and the astra toolbox for advanced processing and reconstruction of tomographic synchrotron data,” *J. Synchrotron Radiation* 23, 842–849 (2016).
3. Pelt, D. M., Batenburg, K. J. & Sethian, J. A., “Improving tomographic reconstruction from limited data using mixed-scale dense convolutional neural networks,” *J. Imaging* 4, 128 (2018).
4. Andrade-Loarca, H., Kutyniok, G., Öktem, O. & Petersen, P., “Deep microlocal reconstruction for limited-angle tomography,” *Appl. Comput. Harmon. Analysis* 59, 155–197 (2022).
5. Koho, S. et al., “Fourier ring correlation simplifies image restoration in fluorescence microscopy,” *Nat. communications* 10, 3103 (2019).
